# Corticosteroid prevents COVID-19 progression within its therapeutic window: a multicentre, proof-of-concept, observational study

**DOI:** 10.1080/22221751.2020.1807885

**Published:** 2020-08-21

**Authors:** Yang Li, Xian Zhou, Tao Li, Shiji Chan, Yiqi Yu, Jing-Wen Ai, Haocheng Zhang, Feng Sun, Qiran Zhang, Lei Zhu, Lingyun Shao, Bin Xu, Wenhong Zhang

**Affiliations:** a Departments of Infectious Diseases, Huashan Hospital, Fudan University, Shanghai, People’s Republic of China; b Department of Tuberculosis, Shanghai Public Health Clinical Center, Fudan University, Shanghai, People’s Republic of China; c Department of Infectious Diseases, Wenzhou Central Hospital, Wenzhou, People’s Republic of China; d Departments of Respiratory Diseases, Zhongshan Hospital, Fudan University, Shanghai, People’s Republic of China; e National Clinical Research Center for Aging and Medicine, Huashan Hospital, State Key Laboratory of Genetic Engineering, School of Life Science, Key Laboratory of Medical Molecular Virology (MOE/MOH) and Institutes of Biomedical Sciences, Shanghai Medical College, Fudan University, Shanghai, People’s Republic of China

**Keywords:** COVID-19, corticosteroids, SARS-CoV-2

## Abstract

Critically ill patients with coronavirus diseases 2019 (COVID-19) are of grave concern. Those patients usually underwent a stage of excessive inflammation before developing acute respiratory distress syndrome. In this study, we test the hypothesis that short-term, low-to-moderate-dose corticosteroids would benefit patients when used in the early phase of excessive inflammation, namely, the therapeutic window. Among a Shanghai cohort and a validation cohort, we enrolled COVID-19 patients showing marked radiographic progression. Short-term, low-to-moderate-dose corticosteroids were considered for them. After identifying the possible markers for the therapeutic window, we then divided the patients, based on whether they were treated with corticosteroids within the therapeutic window, into the early-start group and control group. We identified that the therapeutic window for corticosteroids was characterized by a marked radiographic progression and lactase dehydrogenase (LDH) less than two times the upper limit of normal (ULN). The Shanghai cohort comprised of 68 patients, including 47 in the early-start group and 21 in the control group. The proportion of patients requiring invasive mechanical ventilation was significantly lower in the early-start group than in the control group (10.6% vs. 33.3%, difference, 22.7%, 95% confidence interval 2.6–44.8%). Among the validation cohort of 51 patients, similar difference of the primary outcome was observed (45.0% vs. 74.2%, *P* = 0.035). Among COVID-19 patients with marked radiologic progression, short-term, low-to-moderate-dose corticosteroids benefits patients with LDH levels of less than two times the ULN, who may be in the early phase of excessive inflammation.

## Introduction

As of 26 July 2020, the total number of confirmed coronavirus diseases 2019 (COVID-19) cases has surpassed 15,000,000 cases [1]. Despite the fact that COVID-19 patients have mild symptoms and signs in their early stage, about 8–30% of patients would eventually develop severe illness. Furthermore, the 28-day mortality rate of critically ill patients is over 60% [2]. It thus calls for an urgent need to properly identify high-risk cases that are more likely to deteriorate and consequently impose necessary interventions in the early stage.

**Figure 1.**
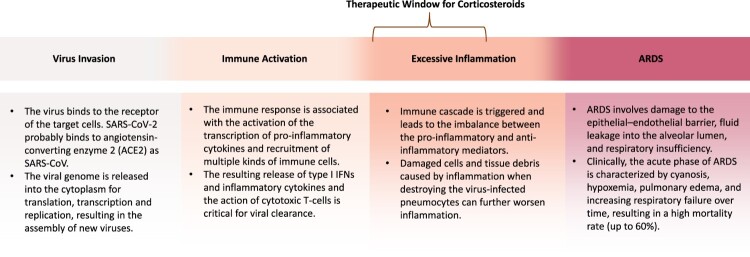
Schema of the pathogenesis of virus-induced ARDS. Balancing virus clearance and host immune response is critical. The early phase of the excessive inflammation is presumed to be the therapeutic window of the corticosteroids [6–11]. Abbreviation: ARDS, acute respiratory distress syndrome.

The use of corticosteroids in the treatment of COVID-19 patients is controversial, considering the inconclusive or even adverse results of previous clinical studies on treating SARS-CoV, MERS-CoV, and other severe respiratory virus infections with corticosteroids [3,4]. Challenging analytical issues with these studies include the selection bias and confounders as physicians tend to use corticosteroids in more severe patients [3,5]. The study population of previous studies might not identify the subjects who could benefit from corticosteroids. According to the currently known molecular mechanisms and pathophysiology data on severe acute respiratory syndrome (SARS), middle east respiratory syndrome (MERS), and influenza patients, critically ill patients usually undergo the following stages: virus invasion, immune activation, excessive inflammatory response, acute respiratory distress syndrome (ARDS), and, in the end, possible recovery or death [6–11]. Although corticosteroids can suppress the inflammatory response, using it too early may suppress the immune activation, thus weaken the viral clearance, while using it too late, the patient is probably too ill to be rescued, as excessive inflammation has progressed, causing ARDS as a result (Figure 1). The therapeutic window of the corticosteroids was presumed to be the early phase of excessive inflammation which varies from person to person and may change dynamically.

We hypothesized that short-term, low-to-moderate-dose corticosteroids therapy in the therapeutic window would most likely benefit the patients. In the study, our first step was to identify possible markers for this therapeutic window; the second step was to demonstrate that patients within the therapeutic window veritably benefit from corticosteroids therapy; the third step was to verify that corticosteroids therapy within the treatment window could also benefit the patients in the validation cohort.

## Methods

### Study population and design

The Shanghai cohort enrolled adult COVID-19 patients who were admitted to Shanghai Public Health Clinical Center. Between 20 January 2020 and 13 February 2020, a total of 311 patients were hospitalized and underwent routine laboratory tests and radiologic examinations. Stably mild patients were given systemic supportive therapy and corticosteroid was not introduced because of potential risks. Patients were considered eligible for corticosteroids once chest radiology examinations suggested they were at risk of progression to ARDS. Therapeutic treatment usually consisted of 40–80 mg/d (0.75–1.5 mg/kg/d) of methylprednisolone for 3 days, then was tapered to 20 mg/d, with a total treatment period of less than 7 days. Corticosteroids might also be used as rescue treatment in more severe or critically ill patients, and its dosage and course were generally individualized based on clinicians’ experiences and patients’ conditions.

Rather than determining the effectiveness of corticosteroids therapy among all patients, this study aimed to evaluate whether corticosteroids therapy would benefit the patients in the therapeutic window, i.e. the early stage of excessive inflammation. Therefore, the first step of this study was to identify possible markers for disease progression. Although radiographic progression could help clinicians to identify patients who undergo excessive inflammation, the stage before ARDS, it might fail to accurately distinguish different phase of excessive inflammation. Thus, we needed a dynamic indicator that could reflect the patient’s phase of excessive inflammation, whether early or late. We evaluated all the laboratory parameters that we monitored closely, determining the association between variates and diseases severity, trying to find a certain indicator that on par with the disease progression. Based on this, eligible patients would be divided into two groups according to the timing of corticosteroids therapy: the early-start group, as patients were treated with corticosteroids at the early phase of excessive inflammation, and the control group, as patients were treated with corticosteroids at the late phase of excessive inflammation, later as rescue treatment, or did not take corticosteroids.

Oral informed consent was obtained from each subject and the study protocol conforms to the ethical guidelines of the 1975 Declaration of Helsinki as reflected in an approval by the hospital’s human research committee.

### Validation cohort

We separately performed validation analysis using a retrospective cohort of 187 consecutive adult patients with COVID-19, including 74 patients admitted to one ward of Wuhan *Jin yin-tan* Hospital from 6 January to 10 February 2020, 55 patients admitted to one ward of Wenzhou Centre Hospital from 6 January to 10 February 2020, and 58 patients admitted to one ward of Wuhan Guanggu Hospital from 10 February to 26 February 2020. The methodology of the sample identification was identical to those applied in the Shanghai cohort.

### Clinical data collection and assessment

The clinical data were obtained from the patients’ medical records in the hospital database including age, gender, comorbidities, signs and symptoms, laboratory and radiographic findings, and treatment measures.

COVID-19 patients were sorted according to WHO interim guidance [12] based on their clinical syndromes. This study used the following brief definition: mild patients were defined as those with uncomplicated illness or mild pneumonia; severe patients were defined as those with severe pneumonia; critically ill patients were defined as those with ARDS, sepsis and septic shock.

### Radiographic assessment

Patients were enrolled if they had marked radiographic progression, which was defined according to the following criteria: a rapid progression of pneumonia defined by size increasing by more than 50% with involvement of one-third of the lung fields within 48 h or the presence of extensive ground-glass opacity involving more than half of the lung fields. Radiographic assessments were performed with the simultaneous consensus of two radiologists who were blinded to the patient’s clinical information except the diagnosis of COVID-19.

### Outcome measures

The primary outcome was the proportion of patients requiring invasive mechanical ventilation, with data censored at 26 March 2020. Thus, we did not enroll patients on invasive mechanical ventilation at admission, regardless of whether corticosteroids were used during hospitalization.

The secondary outcome was the safety of corticosteroids. The complications of corticosteroid therapy were monitored and noted, including secondary infection, osteoporosis, psychosis, delayed clearance of viral, and avascular necrosis. Blood glucose monitoring was performed closely among patients with diabetes.

### Statistical analysis

SPSS software, version 22.0 (IBM) was used for the statistical analysis. Continuous variables were summarized by means ± standard deviation (SD) and were compared with the *t* tests. For categorical variables, the chi-square test and Fisher’s exact test were used for group comparisons. For the primary outcome, the difference and its 95% confidence interval (CI) was calculated [13]. Multivariate logistic analysis was used for finding the independent risk factors associated with the primary outcome. Spearman’s correlation analysis was used for measuring the degree of associations. Two-tailed *P* values of less than 0.05 were considered to be statistical significance.

## Results

### Identification the predictive markers for disease progression

Among 311 patients enrolled, SARS-CoV-2 pneumonia progressed to severe diseases in 11 of them, and 16 patients developed critically illness (including 1 patient with invasive mechanical ventilation before admission), while the other 284 patients remained stably mild during the follow-up.

**Figure 2.**
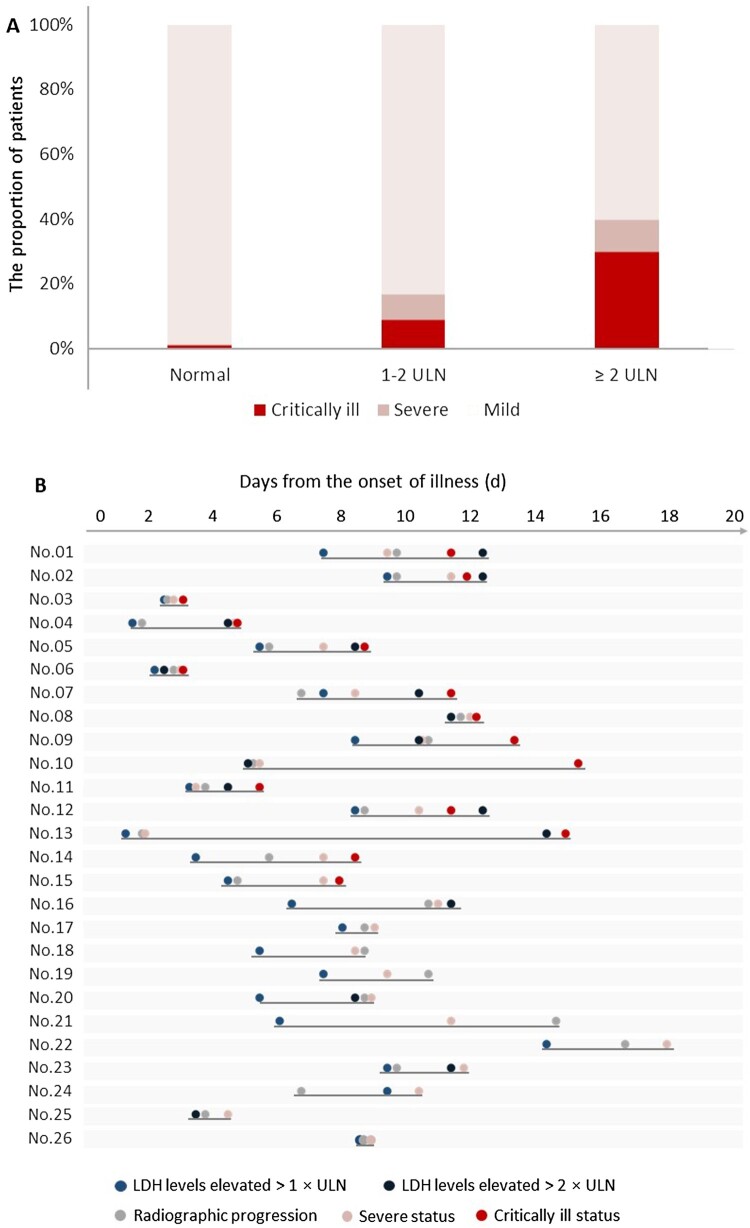
The relationship of lactic dehydrogenase levels and disease severity. (A). Critically ill patients were more common in those with lactic dehydrogenase levels more than two times the ULN. (B). Timeline of clinical events in 26 severe and critically ill cases among Shanghai COVID-19 patients.

To determine the potential predictive markers for disease progression, we quantified the strength of the association strength between several clinical laboratory parameters at admission and disease severity (Supplement Table S1). Among all parameters, the association between lactase dehydrogenase (LDH) level and disease severity was strongest (Rho, 0.338; *P* < 0.001). We found that patients with LDH levels > 2 times the upper limit of normal (ULN) at admission, comparing with those with LDH < 2ULN, were more likely to develop critical illness (30% [3/10] vs. 4.3% [13/301], *P* = 0.011) (Figure 2(A)). Furthermore, when analysing the exact time of the LDH elevation, radiographic progression and clinical condition deterioration, we found that the LDH elevated (to above ULN) earlier than disease progression, on an average of 2.2 days before disease progressed to severe illness (95% CI, 1.6–2.9, *P* < 0.001) and 3.9 days before disease progressed to critical illness (95% CI 2.0–5.9, *P* < 0.001). Similarly, marked radiographic progression was observed to have an average of 0.7 days (95% CI 0.1–1.3, *P* = 0.021) and 3.5 days (95% CI 1.5–5.5, *P* = 0.002) earlier before clinical disease progression to severe and critically ill status, respectively (Figure 2(B)).

Therefore, we thought that serum LDH level might be a valuable predicative marker for diseases progression [14], more importantly, could demonstrate the potential for depicting the spectrum of severity of diseases. Patients who with extremely high LDH levels, i.e. >2ULN, were probably at the late stage of excessive inflammatory response and therefore, had missed the therapeutic window of corticosteroids. Thus, we aimed to determine the benefits of corticosteroids when administered at the early stage of excessive inflammatory response that was characterized by marked radiographic progression and LDH < 2ULN.

### Baseline characteristics of the Shanghai cohort

**Figure 3.**
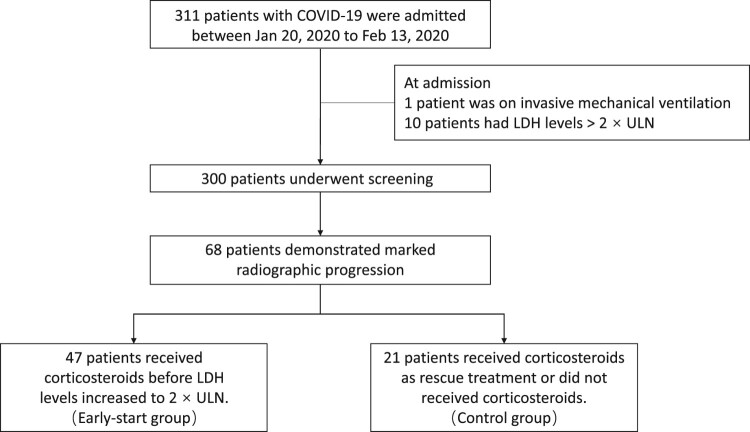
Flow chart of the Shanghai cohort.

Based on the concept above, we excluded one patient with mechanical ventilation and 10 patients with serum LDH>2ULN at admission from the study. Of the remaining 300 patients, a total of 68 patients showed marked radiographic progression with a median time of 7 days (from the onset of illness). Among them, 47 patients were divided into the early-start group as they received corticosteroids before LDH levels increased to 2 times the ULN while the other 21 were divided into the control group including those who received corticosteroids after LDH>2ULN or those who did not received corticosteroids during the whole course (Figure 3).

For the entire Shanghai cohort, the mean age was 58.0 ± 16.5 years. 42 patients (62%) were male. Baseline demographics and clinical characteristics were comparable between two groups (Table 1), as well as the laboratory findings (Table 2). All patients showed radiographic abnormalities that were consistent with SARS-CoV-2 pneumonia at admission and the mean duration from the onset of illness to marked radiographic progression did not differ between two groups (7.6 days vs. 7.1 days, *P* = 0.639).

**Table 1.** Baseline characteristic, symptoms, and comorbidities of patients in the Shanghai cohort.

	Early-start group (*n* = 47)	Control group (*n* = 21)	*P* value
Age, years			0.744
Mean ± standard deviation	57.6 ± 15.5	59.0 ± 18.3	
Median, range	63 (23–84)	64 (24–88)	
Sex, male	30 (63.8)	12 (57.1)	0.600
Symptoms			
Fever	42 (89.4)	19 (90.5)	0.889
Cough	24 (51.1)	11 (52.4)	0.920
Expectoration	10 (21.3)	9 (42.9)	0.067
Fatigue	11 (23.4)	3 (14.3)	0.390
Sore throat	1 (2.1)	1 (4.8)	0.525
Dyspnoea	1 (2.1)	2 (9.5)	0.170
Chest tightness	2 (4.3)	2 (9.5)	0.582
Diarrhoea	2 (4.3)	1 (4.8)	1.000
Headache	2 (4.3)	1 (4.8)	1.000
Chronic medical illness			
Hypertension	15(31.9)	9 (42.9)	0.383
Coronary heart disease	4(8.5)	0(0%)	0.303
Cerebrovascular disease	1 (2.1)	1 (4.8)	0.525
Diabetes mellitus	6 (12.8)	4 (19.0)	0.499
Autoimmune disorders	1(2.1)	0(0%)	1.000

Data are shown as *n* (%) unless specified otherwise.

**Table 2.** Laboratory findings of patients in the Shanghai cohort at admission.

	Early-start group (*n* = 47)	Control group (*n* = 21)	*P* value
Blood routine and lymphocyte classification			
White blood count, ×10^9^/L	4.6 ± 1.6	5.2 ± 1.8	0.199
Neutrophils, ×10^9^/L	3.3 ± 1.6	3.7 ± 1.1	0.452
Lymphocytes, ×10^9^/L	0.89 ± 0.3	1.0 ± 1.1	0.671
CD4 positive cell, cell/µL	312.3 ± 161.1	239.8 ± 156.3	0.132
CD8 positive cell, cell/µL	193.4 ± 102.0	367.6 ± 868.6	0.348
Haemoglobin, g/L	134.7 ± 14.8	134.5 ± 17.9	0.976
Platelets, ×10^9^/L	160.4 ± 55.0	148.0 ± 32.7	0.343
Blood biochemistry			
Alanine aminotransferase, U/L	29.4 ± 20.5	36.4 ± 24.1	0.223
Aspartate aminotransferase, U/L	33.5 ± 15.3	41.6 ± 22.5	0.087
Albumin, g/L	38.6 ± 4.2	38.4 ± 3.6	0.852
Creatine, μmol/L	76.1 ± 34.8	72.6 ± 19.7	0.678
eGFR, mL/(min×1.73 m^2^)	103.7 ± 29.1	97.6 ± 19.3	0.384
Creatine kinase, U/L	195.1 ± 233.5	328.9 ± 627.7	0.365
Troponin T, ng/mL	0.036 ± 0.037	0.042 ± 0.036	0.620
Lactate dehydrogenase, U/L	289.3 ± 78.9	323.9 ± 92.1	0.117
NT-proBNP, pg/mL	163.5 ± 359.7	208.1 ± 475.1	0.671
Coagulation function			
Prothrombin time, s	13.4 ± 0.60	13.7 ± 1.18	0.187
APTT, s	41.5 ± 4.2	45.2 ± 8.6	0.069
FDP, μg/mL	2.1 ± 3.9	1.60 ± 1.38	0.570
d-dimer, μg/mL	1.25 ± 2.98	0.77 ± 0.54	0.465
Infection-related parameters			
C-reactive protein, mg/L	35.8 ± 38.0	38.8 ± 28.7	0.931
Procalcitonin, ng/mL	0.10 ± 0.33	0.13 ± 0.14	0.941
ESR, mm/h	65.4 ± 38.9	57.1 ± 32.2	0.258

Notes: Data are shown as mean ± standard deviation. APTT, Activated partial thromboplastin time; eGFR, estimated glomerular filtration rate; ESR, erythrocyte sedimentation rate; FDP, Fibrinogen degradation products; NT-proBNP, N-terminal pro-B-type natriuretic peptide.

### Clinical outcomes in the Shanghai cohort

**Figure 4.**
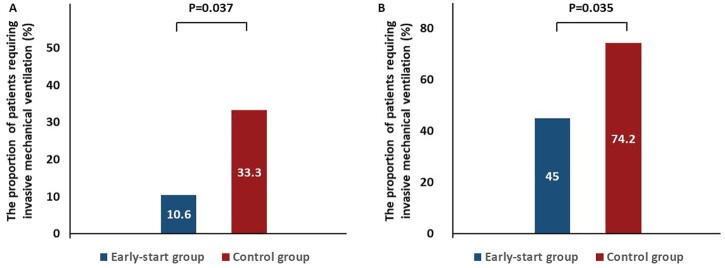
The proportion of the patients with requirement of invasive mechanical ventilation in the Shanghai cohort (A) and the validation cohort (B).

The proportion of patients requiring invasive mechanical ventilation was significantly lower in the early-start group (10.6% [5/47]) than in the control group (33.3% [7/21], *P* = 0.037; Figure 4(A)), with a difference in proportion of 22.6% (95% CI, 2.6–44.8%) between the two groups. The duration from onset of symptoms to invasive mechanical ventilation did not significantly differ between two groups (10.4 days vs. 9.8 days, *P* = 0.873).

Regarding the safety profiles, by February 27, all patients were SARS-CoV-2 RT–PCR negative except one patient in the early-start group and three patients in the control group (two cases with the rescue use of corticosteroids and one case without the use of corticosteroids). Three patients with rescue using of corticosteroids developed secondary infection: two developed carbapenem-resistant *Acinetobacter baumannii* infection and one developed Staphylococcus haemolyticus bacteraemia. One patient in the early-start group developed venous catheter-associated candidemia and acute cerebral infarction.

### Assessment in the validation cohort

A total of 187 patients were admitted and were evaluated for eligibility of the study. As results, the validation cohort was composed of 51 eligible patients (20 in the early-start group and 31 in the control group; Supplemental Figure S1). The mean (SD) age of the early-start group was 55.3 (11.0) years and of the control group was 65.7 (14.5) years (*P* = 0.010, Supplemental Table S2). Other baseline characteristics, including symptoms, comorbidities and laboratory findings, did not significantly differ between two groups (Supplemental Table S2, S3).

During follow-up, 9 of the 20 patients in the early-start group were observed to have SARS-CoV-2 pneumonia progression, eventually requiring invasive mechanical ventilation, which was significantly less common compared with the control group (45.0% [9/20] vs. 74.2% [23/31], *P* = 0.035) (Figure 4(B)). Multivariate logistic analysis (adjusted for age) showed that the early use of corticosteroids independently reduced the risk of disease progression (Odds hazards: 0.201, 95% CI: 0.048–0.846; *P* = 0.029).

In the early-start group, secondary infection occurred in two patients, one of whom developed *Candida albicans* bloodstream infection and one of whom was clinically diagnosed with aspergillosis. Hyperglycemia with glucose value of 500 mg/dL was reported in one patient, while ketosis or ketoacidosis was not developed. In contrast, three patients with rescue use of corticosteroids reported candidiasis and two patients without corticosteroids use reported *Klebsiella pneumoniae* infection. Two patients in the early-start group, one with rescue use of corticosteroids, and four without use of corticosteroids remained SARS-CoV-2 PCR positive with throat swabs.

## Discussion

This study is the first multicenter clinical study addressing the issue of the therapeutic effects of corticosteroids on COVID-19 cases. By identifying the therapeutic window in the Shanghai cohort and confirming our theory in the validation cohort, we demonstrated that patients in the early phase of excessive inflammation could benefit from the short-term, low-to-moderate-dose corticosteroids therapy.

**Figure 5.**
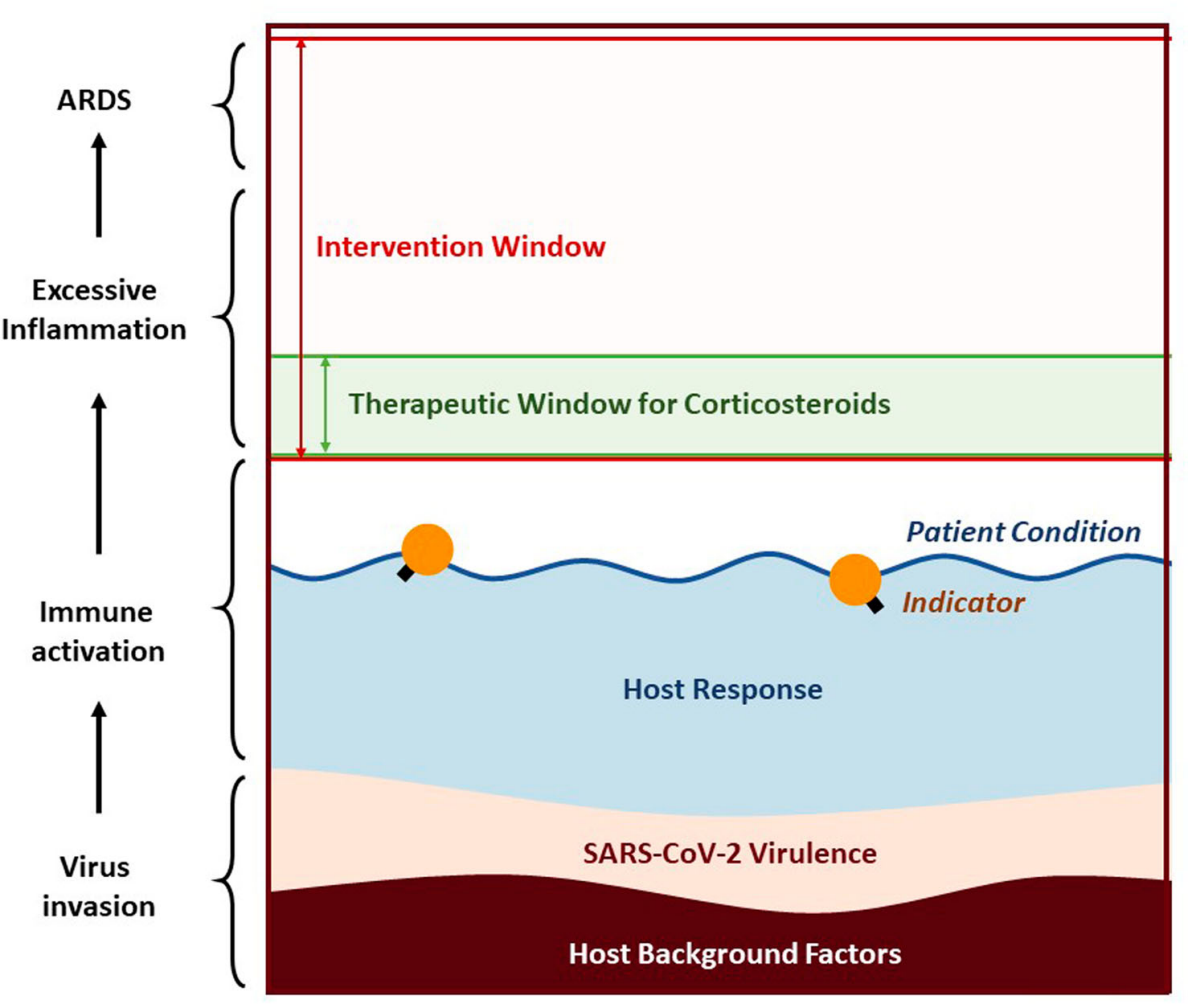
Theoretical schema describing SARS-CoV-2 infection and the therapeutic window for corticosteroids. The patient’s condition (water level in the figure, shown in the navy-blue line) is the result of a combination of many factors. For COVID-19 patients, these factors should at least include host background (brown), SARS-CoV-2 virulence (beige), and host response (sky blue). Based on the knowledge of SARS, MERS, and other severe respiratory virus infections, host response plays a key role in the disease progression towards ARDS. The intervention window (pink) of the disease is the interval from when the patient needs medical intervention to when the patient cannot be rescued by any available measures. Theoretically, different treatments have different therapeutic windows. For corticosteroids, its therapeutic window should be the early phase of excessive inflammation in COVID-19 patients. Whether initiating the corticosteroids therapy should base on the relationship between the patient condition (blue line) and the therapeutic window (green). For mild patients, the level of patient condition would be stably lower than the therapeutic window for corticosteroids, who may even never require any medical interventions. For the patients with high-risk factors such as advanced age, the area in brown would be larger and the level of blue line rose correspondingly, resulting in a narrower space to the intervention window and greater possibility of exceeding the therapeutic window for corticosteroids. An Ideal indictor is what exactly reflects the patient condition just as the buoy (orange icon) on the river.

The timing applying corticosteroids is critical. Based on molecular mechanisms and physiologic data, dysregulated inflammation has played a significant role in the progression of ARDS [6–11]. Theoretically, corticosteroids should be able to suppress inflammation. However, corticosteroids are obviously not panacea. We should not expect corticosteroids to work in the late phase of excessive inflammatory response, while huge amounts of pro-inflammatory cytokines have formed. Nor shall we expect corticosteroids to revive the damage caused by extensive inflammation, especially when developing into ARDS, which is incurable for the time being [15]. We consider this as the reason that many studies reached negative conclusions on the use of corticosteroids in the intensive care unit (ICU) or critically ill patients [5,16–18]. These patients were probably having an advanced stage of disease too late to be rescued by corticosteroids. On the other hand, for mild patients, we think it is inappropriate to use corticosteroids with no evidence of disease progression. Most patients’ immune system was activated in a proper way that would never produce excessive cytokines and they would recover from the infection without further intervention (Figure 5). Therefore, a therapeutic window for corticosteroids should be established.

Our hypothesis is that the therapeutic window is at the early stage of excessive inflammatory response, as patients may deteriorate since then, and corticosteroids are probably effective at that point. However, how to determine the beginning and end of this therapeutic window is the tricky part. A constant marker, such as 72 h after symptom onset [19] or the day of ICU admission [16], may not be a good choice as clinical conditions are constantly changing and vary individually. An ideal indicator should be able to dynamically reflect the patient’s condition, and its value should be completely parallel to the disease progression. Thus, we focused on the laboratory parameters for their objectivity, changeability, and scalability. For those who have high-risk host factors, we are likely to obtain a narrower therapeutic window, hence, closely monitoring against time is urgent for their early intervention (Figure 5).

As shown in our validation cohort, patients in the early start group were much younger than the control group. However, the mean age between patients requiring invasive mechanical ventilation shows no difference, suggesting that age was not related to the outcome in our study. This finding inspired us to assume that, younger patients may have a wider therapeutic window, resulting a higher possibly of receiving corticosteroids in time. While older patients are more vulnerable to hypoxia or other injuries, their LDH is more likely to exceeds 2 times the ULN. The risk of advanced age during the course of the disease is ultimately reflected in LDH levels.

Recently, RECOVERY study showed that dexamethasone reduced 28-day mortality among those who received either invasive mechanical ventilation or oxygen alone but not who did not need respiratory support [20]. The results from RECOVERY study identified another therapeutic window of dexamethasone, which was similar but wider than that of our study. Both RECOVERY study and our study emphasized that corticosteroids could not benefit all COVID-19 patients and the therapeutic window of corticosteroids should be established. The value of both studies is to identify the subgroup of patients within the therapeutic window where the benefit of corticosteroid outweighs its risk. In this study, the therapeutic window of corticosteroids was characterized by marked radiographic progress with LDH levels of less than 2ULN, which merits further investigation. Marked radiographic progression is usually a sign of disease deterioration according to clinical experience, but it is probably delayed when taking the theoretical beginning of the excessive inflammation into account, for imaging itself can be seen as a part of the consequence of excessive inflammation. Although LDH was the best indicator of disease status among all the indicators we are able to evaluate, our methods could be optimized further. It should be noted that imaging and LDH levels combined is just one of the ways to identify the therapeutic window. By combining other indicators, such as lymphocyte count, CD4 cell count, d-dimer, and even genetic susceptibility typing in the future, we believe that the patients subgroup who could benefit from corticosteroids therapy will be targeted more accurately.

Adverse reactions of corticosteroids have been a great concern. Like other drugs, adverse reactions of corticosteroids cannot be completely prevented. Even so, corticosteroids are still the first-line treatment for many other diseases. For these diseases, the benefits of corticosteroids far outweigh the risks and adverse reactions. Therefore, we should pay more attention to the benefit-risk ratio while consider applying corticosteroids. The benefits can be further fortified after finding a corticosteroids-suitable population, as we have demonstrated; while the risk of adverse reactions can be reduced by decreasing the dose and duration of corticosteroids. For the early intervention group, our starting dose of corticosteroids was methylprednisolone 40–80 mg/d (0.75–1.5 mg/kg/d). Generally, the dosage was reduced after 3 days of treatment, and the total course of treatment did not exceed 7 days. Adverse reactions to corticosteroids in the early-start group were no more than in the control group. The data on adverse reactions in our study further supports the strategy of the early usage of low-to-moderate-dose corticosteroids in high-risk patients.

Corticosteroids may delay virus clearance in COVID-19 patients, which becomes another major concern applying corticosteroids while the relevant data is still limited. Nelson Lee et al. [21] conducted a randomized controlled trial of 16 patients with SARS; the median time for SARS-CoV to become undetectable in plasma was 12 days (11–20 days) in the hydrocortisone group versus 8 days (8–15 days) in controls. It should be noted, however, that this cohort only included non-severe patients with symptom onset less than 5 days, while excluding patients with comorbidities or with oxygen saturation < 90% without supplemental oxygen therapy. As a result, the proportion of patients who would develop severe illness is very low in this cohort. Data on the effects of corticosteroids on viral clearance in this group of patients were very valuable; however, it is inappropriate to evaluate the benefit-risk ratio of corticosteroids in this cohort. Potentially delayed viral clearance should not mitigate the benefits of corticosteroids on preventing disease progression. The relationship between virus clearance time, viral load, and prognosis is unclear in COVID-19 patients.

This study has several limitations. First, the generalizability is limited because of the nature of the retrospective study, although we have validated our results in another cohort. Second, our strategy of finding suitable corticosteroids in the early stage based requires frequent CT examinations and LDH tests, which might be difficult in the areas with limited resources. However, considering the severe-critically ill case 28-day mortality rate of 61.5% [2], and huge medical resource investment during treatment of critically ill patients, we believe that early screening of high-risk patients for early intervention is worthwhile. Third, as a proof-of-concept study, what we found was the patients who would benefit from corticosteroids but not who would benefit most. We are very much looking forward to more precise and accurate corticosteroids therapeutic windows in the future.

## Conclusion

Among COVID-19 patients with marked radiologic progression, short-term, low-to-moderate-dose corticosteroids could benefit patients with LDH levels of less than two times ULN, who may be in the early phase of excessive inflammation. With closely radiographic assessment and LDH monitoring, clinicians would be able to identify more patients in the therapeutic window and apply corticosteroids in time to prevent them from disease progression.

## Ethics approval and consent to participate

An ethical approval was obtained from the Ethics Review Board of the Huashan Hospital, Fudan University (ethics approval registration number: KY2020-689). All patients who participated in the study gave written informed consent.

## Supplementary Material

Supplemental_Table.docx

## Data Availability

The data that support the findings of this study are available from the corresponding author on reasonable request.

## Abbreviations

**ABBREVIATIONS:** ARDS: acute respiratory distress syndrome; CI: confidence interval; COVID-19: coronavirus diseases 2019; LDH: lactase dehydrogenase; MERS: middle-east respiratory syndrome; SARS: severe acute respiratory syndrome; ULN: upper limit of normal
