# Supplementary material for: Corticosteroid prevents COVID-19 progression within its therapeutic window: a multicentre, proof-of-concept, observational study
Source: Emerg Microbes Infect. 2020 Aug 21;9(1):1869–77. doi: 10.1080/22221751.2020.1807885 (PMC7473313; doi:10.1080/22221751.2020.1807885)
Supplement: Supplemental_Table.docx [file TEMI_A_1807885_SM1147.docx]

**Supplement Table 1**. The spearman’s correlation between the laboratory parameters and disease severity in the Shanghai Cohort.

|  | **Spearman’s rho** | | **P value** |
| --- | --- | --- | --- |
| **Blood routine and lymphocyte classification** | |  |  |
| White blood count | 0.028 | | 0.619 |
| Neutrophils | 0.099 | | 0.082 |
| Lymphocytes | -0.247 | | **0.001** |
| CD4 positive cell | -0.311 | | **<0.001** |
| CD8 positive cell | -0.236 | | **<0.001** |
| Hemoglobin | 0.031 | | 0.588 |
| Platelets | -0.114 | | **0.044** |
| **Blood biochemistry** |  | |  |
| Alanine aminotransferase | 0.089 | | 0.115 |
| Aspartate aminotransferase | 0.249 | | **<0.001** |
| Albumin | -0.275 | | **<0.001** |
| Creatine | 0.140 | | **0.014** |
| eGFR | -0.181 | | **0.001** |
| Creatine kinase | 0.311 | | **<0.001** |
| Troponin T | 0.216 | | **<0.001** |
| Lactate dehydrogenase | 0.338 | | **<0.001** |
| NT-proBNP | 0.256 | | **<0.001** |
| **Coagulation function** |  | |  |
| Prothrombin time | 0.064 | | 0.258 |
| APTT | 0.132 | | **0.020** |
| FDP | 0.185 | | **0.002** |
| D-dimer | 0.203 | | **<0.001** |
| **Infection-related parameters** |  | |  |
| C-reactive protein | 0.333 | | **<0.001** |
| Procalcitonin | 0.300 | | **<0.001** |
| ESR | 0.019 | | 0.743 |

Diseases were rated as mild, severe, or critically ill in this study.

**Supplement Table 2** Baseline characteristic, symptoms, and comorbidities of the validation cohort

|  | **Early-start Group**  **(n=20)** | **Control Group**  **(n=31)** | **P value** |
| --- | --- | --- | --- |
| **Age, years** |  |  | **0.010** |
| Mean ± Standard deviation | 55.3±11.0 | 65.7±14.5 |  |
| Median, range | 57 (23-74) | 71 (25-85) |  |
| **Sex, Male** | 12 (60.0) | 24 (77.4) | 0.183 |
| **Symptoms** |  |  |  |
| Fever | 19 (95.0) | 29 (93.5) | 1.000 |
| Cough | 16 (80.0) | 23 (74.2) | 0.633 |
| Expectoration | 6 (30.0) | 13 (41.9) | 0.389 |
| Fatigue | 5 (38.5) | 3 (20.0) | 0.183 |
| Dyspnea | 4 (20.0) | 7 (22.6) | 1.000 |
| Diarrhea | 1 (5.0) | 1 (3.2) | 1.000 |
| Headache | 0 (0.0) | 3 (9.7) | 0.271 |
| **Chronic medical illness** |  |  |  |
| Hypertension | 6 (30.0) | 17 (54.8) | 0.082 |
| Coronary heart disease | 0 (0) | 1 (3.2) | 1.000 |
| Cerebrovascular disease | 0 (0) | 1 (3.2) | 1.000 |
| Diabetes mellites | 0 (0) | 6 (19.4) | 0.070 |
| Malignant tumors | 1 (5.0) | 2 (6.5) | 1.000 |

Data are shown as n(%) unless specified otherwise.

**Supplement Table 3** Laboratory findings of the validation cohort at admission.

|  | **Early-start Group**  **(n=20)** | | **Control Group**  **(n=31)** | | **P value** |
| --- | --- | --- | --- | --- | --- |
| **Blood routine and lymphocyte classification** | |  | |  |  |
| White blood count, ×10^9^/L | 5.45±4.65 | | 6.09±2.84 | | 0.563 |
| Neutrophils, ×10^9^/L | 4.39±4.51 | | 5.07±2.82 | | 0.521 |
| Lymphocytes, ×10^9^/L | 0.70±0.36 | | 0.61±0.36 | | 0.379 |
| Hemoglobin, g/L | 121.9±18.0 | | 125.3±20.1 | | 0.567 |
| Platelets, ×10^9^/L | 153.9±57.6 | | 170.6±56.7 | | 0.328 |
| **Blood biochemistry** |  | |  | |  |
| Alanine aminotransferase, U/L | 51.9±90.5 | | 27.7±15.8 | | 0.179 |
| Aspartate aminotransferase, U/L | 48.3±28.9 | | 41.9±21.8 | | 0.393 |
| Albumin, g/L | 31.3±4.0 | | 29.8±3.1 | | 0.188 |
| Creatine, μmol/L | 77.1±24.6 | | 79.8±27.8 | | 0.730 |
| eGFR, ml/(min×1.73m^2^) | 94.2±21.2 | | 91.2±27.4 | | 0.699 |
| Troponin T, pg/mL | 3.61±5.12 | | 16.1±34.03 | | 0.200 |
| Lactate dehydrogenase, U/L | 342.5±99.9 | | 332.3±89.1 | | 0.714 |
| NT-proBNP, pg/mL | 53.0±49.4 | | 248.7±400.6 | | 0.070 |
| **Coagulation function** |  | |  | |  |
| Prothrombin time, s | 12.7±1.36 | | 26.5±43.8 | | 0.254 |
| APTT, s | 31.3±8.8 | | 34.3±7.9 | | 0.341 |
| FDP, μg/ml | 12.1±15.1 | | 8.2±7.8 | | 0.531 |
| D-dimer, μg/ml | 2.67±5.24 | | 6.30±9.28 | | 0.220 |
| **Infection-related parameters** |  | |  | |  |
| C-reactive protein, mg/L | 74.3±71.4 | | 50.5±38.6 | | 0.188 |
| Procalcitonin, ng/mL | 0.87±2.47 | | 0.18±0.26 | | 0.326 |
| ESR, mm/h | 58.2±17.7 | | 46.5±25.1 | | 0.167 |

Data are shown as mean ± standard deviation.

Abbreviation: NT-proBNP, N-terminal pro-B-type natriuretic peptide; eGFR, estimated glomerular filtration rate; APTT, Activated partial thromboplastin time; FDP, Fibrinogen degradation products; ESR, erythrocyte sedimentation rate.
